# Redox Regulation of Stem-like Cells Though the CD44v-xCT Axis in Colorectal Cancer: Mechanisms and Therapeutic Implications: Erratum

**DOI:** 10.7150/thno.109534

**Published:** 2025-01-09

**Authors:** Huai-Qiang Ju, Yun-Xin Lu, Dong-Liang Chen, Tian Tian, Hai-Yu Mo, Xiao-Li Wei, Jian-Wei Liao, Feng Wang, Zhao-Lei Zeng, Helene Pelicano, Mitzi Aguilar, Wei-Hua Jia, Rui-Hua Xu

**Affiliations:** 1 Sun Yat-sen University Cancer Center, State Key Laboratory of Oncology in South China, Collaborative Innovation Center for Cancer Medicine, Guangzhou, 510060, China.; 2 Department of Molecular and Cellular Oncology, The University of Texas MD Anderson Cancer Center, Houston, TX 77030, USA.; 3 Department of Translational Molecular Pathology, The University of Texas MD Anderson Cancer Center, Houston, TX 77030, USA.

The authors regret some incorrect representative images were accidentally displayed during data organization, including flow cytometry scatter diagram in Figure 1F, Figure 4B, Figure S5B and colony image in Figure 6B. The authors confirm that these corrections do not change the result interpretation or conclusions of the article. The authors apologize for any inconvenience that the errors may have caused.

## Figures and Tables

**Figure 1:**
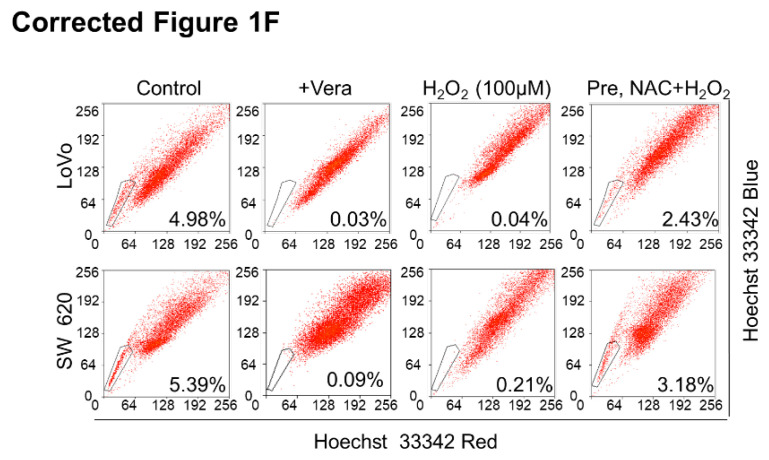
** (F)** Reversion of H_2_O_2_-induced decreases in SP cells following NAC treatment. LoVo or SW620 cells were treated with 3 mM NAC for 2 h, followed by 100 μM H_2_O_2_ for 24 h.

**Figure 4:**
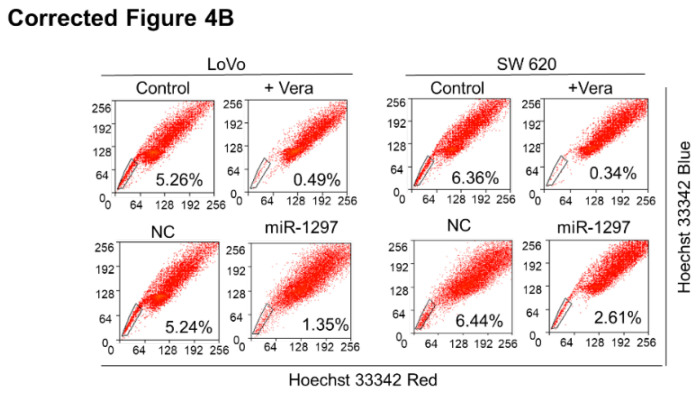
** (B)** CRC SP cell quantification in cells transfected with negative control (NC) or miR-1297 mimic.

**Figure 6:**
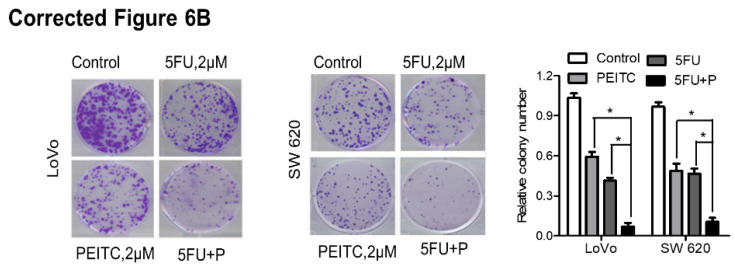
**(B)** LoVo and SW620 cells were incubated with 5FU, PEITC, or both for 2 weeks, and the cell colonies were fixed in formalin, stained with crystal violet, and counted.

**Figure A:**
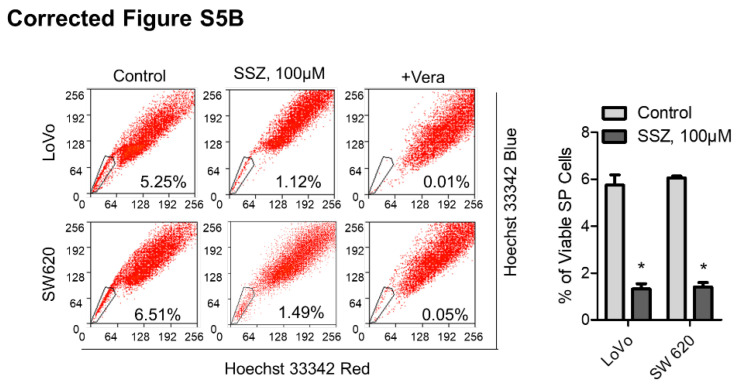
** Correct image for Figure S5B. (B)** CRC cells were pretreated with SSZ (100 μM) for 48 h and then washed and cultured in fresh medium without the drug for 48 h to allow cell death to occur. Viable cells were harvested and then stained with Hoechst 33342 to identify SP cells, and the viable SP cells were quantified.

